# Genetic Biomarkers and Their Clinical Implications in B-Cell Acute Lymphoblastic Leukemia in Children

**DOI:** 10.3390/ijms23052755

**Published:** 2022-03-02

**Authors:** Monika Lejman, Aleksandra Chałupnik, Zuzanna Chilimoniuk, Maciej Dobosz

**Affiliations:** 1 Laboratory of Genetic Diagnostics, Medical University of Lublin, 20-093 Lublin, Poland; 2 Student Scientific Society, Laboratory of Genetic Diagnostics, Medical University of Lublin, 20-093 Lublin, Poland; olachalupnik@op.pl (A.C.); zuzia.chil@gmail.com (Z.C.); macdob.98@gmail.com (M.D.)

**Keywords:** childhood acute lymphoblastic leukemia, ALL, B-ALL, T-ALL, biomarkers

## Abstract

Acute lymphoblastic leukemia (ALL) is a heterogeneous group of hematologic malignancies characterized by abnormal proliferation of immature lymphoid cells. It is the most commonly diagnosed childhood cancer with an almost 80% cure rate. Despite favorable survival rates in the pediatric population, a significant number of patients develop resistance to therapy, resulting in poor prognosis. ALL is a heterogeneous disease at the genetic level, but the intensive development of sequencing in the last decade has made it possible to broaden the study of genomic changes. New technologies allow us to detect molecular changes such as point mutations or to characterize epigenetic or proteomic profiles. This process made it possible to identify new subtypes of this disease characterized by constellations of genetic alterations, including chromosome changes, sequence mutations, and DNA copy number alterations. These genetic abnormalities are used as diagnostic, prognostic and predictive biomarkers that play an important role in earlier disease detection, more accurate risk stratification, and treatment. Identification of new ALL biomarkers, and thus a greater understanding of their molecular basis, will lead to better monitoring of the course of the disease. In this article, we provide an overview of the latest information on genomic alterations found in childhood ALL and discuss their impact on patients’ clinical outcomes.

## 1. Introduction

Cancers constitute the second leading cause of death, after cardiovascular disease, among children in both high- and low-income countries [1]. In countries such as Australia, Ireland, Switzerland, and the United States, the prevalence of cancer in children is estimated at 140–160 per 1 million children [2,3]. In West Asia, these values reach 180 children per million, and in East and Central Africa, even up to 220 children per million. According to the GLOBOCAN 2020 database, among children aged 0–19, leukemia is the most common childhood malignancy worldwide, followed by brain and central nervous system tumors, non-Hodgkin’s lymphoma (NHL), kidney tumors, and Hodgkin’s lymphoma (HL) [4]. A summary made by Namayandeh et al. outlined that leukemia is responsible for 27% of childhood cancers in the United States, 30% in Ireland and France, 33% in Germany, and 35% in Shanghai, China [5]. The importance of acute lymphoblastic leukemia (ALL) is crucial due to the high mortality rate, ranging from 1.3 to 6.3 per 100,000 men and from 1.1 to 3.8 per 100,000 women [6,7].

Leukemia is caused by abnormal changes in the lymphoid line of blood cells that can affect bone marrow, blood, and extramedullary sites. It may cause bone and joint pain, fatigue and weakness, enlarged lymph nodes, pale skin color, bleeding or bruising easily, fever, or infection [8,9]. ALL is the most common type of this cancer and accounts for over 80% of all acute leukemia cases. It can be classified as B-ALL and T-ALL types, with the former accounting for approximately 85% of cases. The peak incidence of ALL in children occurs between the ages of 1 and 4 and then declines sharply during childhood (5–14 years), and adolescence and young adulthood (15–39 years), with a low point between the ages of 25 and 45 [10]. Significant differences between children and adults can also be noticed in the context of 5-year survival. While in children the 5-year overall survival (OS) was 90%, only 25% of adults over 50 years old were alive 5 years after diagnosis [11,12].

**Figure 1:**
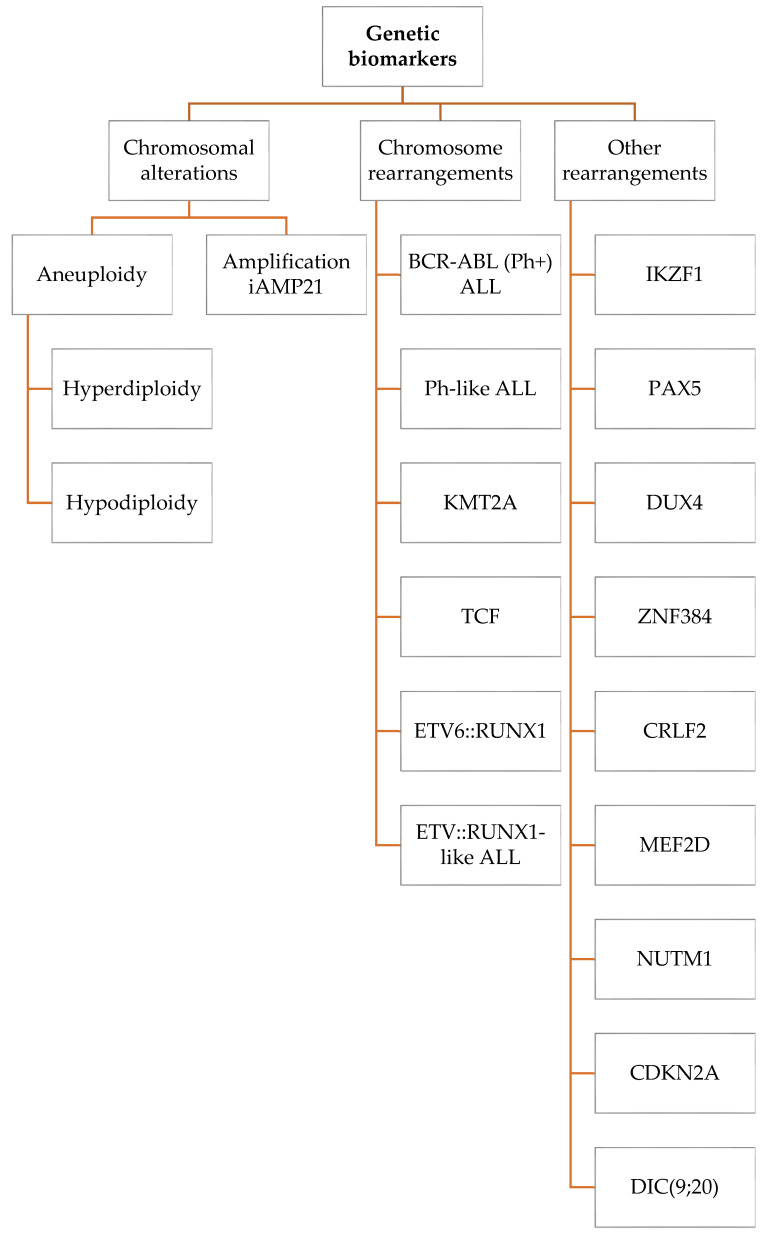
Schematic representation of genetic biomarkers in childhood B-ALL.

In recent years, due to the progression of molecular biology and the development of new technologies, significant advances have been made in understanding the pathophysiology of acute lymphoblastic leukemia. Although most ALL cases occur in healthy individuals, the interaction of environmental risk factors and an inherited genetic susceptibility have been identified in some patients [9]. Epidemiological studies have shown a significant link between the influence of certain factors such as pesticides, ionizing radiation, or infections on the child during pregnancy and early childhood, and the subsequent development of leukemia [13,14,15,16,17,18,19]. Chromosomal and genetic abnormalities also play a significant role in the pathological differentiation and proliferation of lymphoid precursor cells. Acute lymphoblastic leukemia consists of several distinct genetic subtypes characterized by molecular changes such as aneuploidy, chromosome rearrangements, DNA copy number changes, and sequence mutations [20]. The studies indicate a polygenic background of ALL. Genomic alterations are directly involved in the abnormal proliferation of immature lymphoid cells, leading to embryonic and after birth tumor initiation [9]. Mentioned alterations are presented in Figure 1.

Conventional chemotherapy consists of the four important phases: remission induction, consolidation, reinduction (delayed intensification), and continuation (maintenance). Steroids, vincristine, L-asparaginase, cytarabine, methotrexate, and 6-mercaptopurine are administered based on stratified risk classification. Multi-drug pediatric ALL chemotherapy is given in various combinations and in different sequences depending on the treatment protocol. Even though currently almost 80–90% of patients are cured, a significant number of children develop treatment resistance, which results in a poor prognosis [8,21]. With a conventional therapeutic approach, the intensity of the chemotherapy and its toxicity has reached the upper limit. Therefore, in order to improve patient survival and reduce adverse effects, it will be necessary to look for new solutions and approaches. Identification of genetic abnormalities and new ALL biomarkers can help determine the estimated risk of developing or experiencing recurrence of the disease. As a consequence, personalized adjustment of primary therapy to the above conditions may lead to better disease management over time [22].

## 2. Genetic Biomarkers

### 2.1. Chromosomal Alterations

Hyperdiploidy can be divided into two subtypes: high hyperdiploidy (51–65 chromosomes), and low hyperdiploidy (47–50 chromosomes).

High hyperdiploidy (HeH) is present in up to 30% of children and 10% of adults with ALL [23]. In more than 50% of hyperdiploid BCP-ALL patients, the most frequent gained chromosomes are X, 4, 6, 10, 14, 17, 18, and 21. High hyperdiploidy was first observed in 1978 as an anomaly associated with favorable prognostic factors [24]. HeH, in connection with other clinical features, such as the median age of 4 years, or low white blood cell count (WBC) at the time of diagnosis, results in an overall survival rate of approximately 90% [25]. These conclusions were confirmed 10 years later in a follow-up series [26]. Studies have shown that the gain of specific chromosomes is associated with improved outcome. The presence of the triple trisomy (simultaneous gain of chromosomes +4, +10, and +17) is currently used by the Children’s Oncology Group (COG) as a prognostic factor of a very low risk of relapse [27]. In contrast, the UK Medical Research Council ALL97/99 randomized trial showed favorable outcomes for patients with high hyperdiploidy +18 [28]. Some studies report that extra copies of chromosomes +5 and +20 are associated with poorer prognosis [29,30]. A recent retrospective analysis by Amir Enshaei and his colleagues provides information on the UKALL high hyperdiploidy prognostic profile. Two trial datasets were used during the analysis, as a discovery cohort (UKALL 97/99) and a validation cohort (UKALL2003). The good risk profile included karyotypes with +17 and +18 or +17 or +18 in the absence of +5 and +20, and its prognostic effect was independent of minimal residual disease. However, its integration may improve it. This integrated approach taking into account key risk factors may enhance risk stratification in childhood acute lymphoblastic leukemia [31]. Children with HeH ALL respond well to standard chemotherapy regimens and show better treatment outcomes compared to non-hyperdiploid pediatric patients [27,28,29]. This may be because HeH cells are particularly sensitive to methotrexate, which is one of the primary drugs used in modern treatment protocols [32,33,34]. Studies have reported that hyperdiploid lymphoblasts can accumulate high levels of methotrexate polyglutamates (MTXPGs). This ability of them may contribute to the increased sensitivity to the cytotoxic effects of methotrexate, which would explain the good results of therapy with this drug [35]. Mutations targeting genes encoding histone modifiers (*CREBBP*, *WHSC1*, *SUV420H1*, *SETD2*, and *EZH2*) and the RTK-RAS pathway (*FLT3*, *NRAS*, *KRAS*, and *PTPN11*) are common in patients with high hyperdiploidy. This observation could be used to develop new targeted therapies to improve the prognosis of pediatric patients with ALL and high hyperdiploidy [24]. Low hyperdiploidy occurs in approximately 10–11% of children and 10–15% of adult patients with acute lymphoblastic leukemia [36]. Its incidence increases with age. In contrast to high hyperdiploidy, this subtype is associated with an unfavorable prognosis. Studies show that patients with low hyperdiploidy have a shorter survival period [37].

Hypodiploidy is defined as the loss of one or more chromosomes, and it is a rare cytogenetic anomaly in ALL. Hypodiploid karyotype occurs below 7% in children and adults with B-ALL. Most cases of hypodiploidy (80%) present 45 chromosomes, and they are classified as near-diploid ALL, often containing dicentric chromosomes, e.g., dic(9;20), and their clinical outcome is not as poor as typical hypodiploidy. In most studies and treatment protocols, hypodiploid B-ALL is defined as ≤44 chromosomes. It can be divided into three subtypes: near haploidy with 24–31 chromosomes, low hypodiploidy with 32–39 chromosomes, and high hypodiploid with 40–44 chromosomes. Patients with hypodiploid B-ALL present with a lower diagnostic WBC than patients with non-hypodiploid B-ALL [38,39,40]. Near-haploidy occur in approximately 0.5% of pediatric B-ALL patients. In near-haploid ALL, retention of disomies X/Y, 8, 10, 14, 18, and 21 can be observed [41]. Studies show that in this subtype, the most common genetic alterations involve RAS signaling (*NRAS*—15% of patients; *FLT3*—9% of patients; *KRAS*—3% of patients; and *PTPN11*—1.5% of patients) and receptor tyrosine kinase (RTK), which occurs in 70.6% of cases. In addition, *IKZF3* (13.2%) and histone cluster at chromosome 6p22 (19.1%) deletions are frequently observed [42]. More than 44% of patients with near-haploid B-ALL present focal deletions or point mutations in the *NF1* gene. Furthermore, alterations in *PAG1* (mostly deletions) have been reported in 10% of patients with near-haploid B-ALL [43]. Other often-described abnormalities reported in near-haploid B-ALL include *CDKN2A/B*, *RB1*, and *PAX5*, and more than 31% patients have deletion or insertion–deletion or point mutations in the *CREBBP* gene. At lower frequencies (<5% of patients) point mutations in *EP300* and *EZH2* are also observed in near-haploid B-ALL [42]. Low hypodiploidy is observed in 0.5% of children and in approximately 4% of adult patients, and its frequency increases with age [38]. The retained disomies in this subtype mainly consist of X/Y, 1, 5, 6, 8, 10, 11, 14, 18, 19, 21, and 22 chromosomes [41]. In low hypodiploidy cases, the alterations most often involve genes such as *TP53* (91.2%), *RB1* (41.2%), *IKZF2* (HELIOS; 52.9%), and *CDKN2A/2B* (20%) [42]. *TP53* mutations are most missense mutations in exons 5–8, affecting the DNA binding domain and the nuclear localization sequence. Moreover, *TP53* mutations are frequently identified in non-tumor cells in 50% of the cases of pediatric low-hypodiploid B-ALL, implying that these cases may be a manifestation of Li–Fraumeni syndrome or other germline TP53 cancer-predisposing mutations [44]. Mutations in *CREBBP* are detected in 60% of patients with low-hypodiploid B-ALL. Patients with hypodiploid ALL are associated with poor prognosis. Event-free survival (EFS) rates are 25–40% for near-haploid ALL and 30–50% for low hypodiploid ALL in pediatric patients. OS rates for children with hypodiploid ALL are 35–50% [28,41,45]. Due to poor treatment outcomes, new treatment regimens are still being sought for patients with hypodiploid ALL. It is still unclear whether hematopoietic stem cell transplantation in first complete remission (CR1) is of benefit [46]. Recent studies suggest the potential possibility of treating children with hypodiploid ALL with intensive chemotherapy due to the observation that negative measurable residual disease (MRD) status at the end of induction treatment is associated with improved EFS in these patients [43]. Activation of PI3K and Ras-signaling is observed in hypodiploid ALL cells. Therefore, PI3K inhibitors may potentially represent a new therapeutic option in this subtype of leukemia [42].

Furthermore, chimeric antigen receptor (CAR) T-cell and monoclonal antibodies or bi-specific T-cell engagers (BiTE), such as inotuzumab and blinatumomab, are the main immunotherapy approaches currently in use to treat hypodiploid B-ALL with <40 chromosomes [47].

In addition to near haploidy and low hypodiploidy, there is also so-called masked hypodiploid ALL, which is characterized by a hyperdiploid karyotype resulting from reduplication of the hypodiploid genome [48]. Masked hypodiploidy represents an important diagnostic challenge for diagnostics and clinicians. Doubled low-hyperdiploid karyotypes most commonly show tetrasomy for chromosomes +1, +8, +10, +11, +18, +19, +21, and +22, while doubled near-haploid karyotypes most commonly show tetrasomy for chromosomes +14, +18, +21, and X/Y [49]. As hyperdiploidy with more than 50 chromosomes is usually associated with a favorable prognosis, it is important to ascertain whether the patient has true hyperdiploidy or masked hypodiploid ALL with a far less favorable prognosis [48]. A single nucleotide polymorphism (SNP) array can identify masked hypodiploid clones, especially detect loss of heterozygosity (LOH), which is a very characteristic feature for masked hypodiploidy. Some authors have recently provided algorithms for the proper distinction between masked hypodiploidy from high-hyperdiploid B-ALL using SNP-arrays and concentrating on specific chromosome gains, such as chromosomes 1, 7, and 14 [38].

Intrachromosomal amplification of chromosome 21 is found in approximately 2% of pediatric patients with BCP-ALL [50]. iAMP21 was originally identified as a distinct cytogenetic subgroup of childhood ALL in 2003 [51]. Patients are older children (median age 9 years, range 2–30 years, compared to median age of 2–5 years for other childhood ALL subtypes) and they mostly have a low white cell count [52]. The iAMP21 chromosome is an abnormal version of chromosome 21, containing multiple regions of gain, amplification, inversion, and deletion. The abnormal chromosome 21 occurred through Breakage–Fusion–Bridge (BFB) cycles followed by chromothripsis, defined as catastrophic shattering and disorganized repair of a single chromosome or chromosomal region within a single cell cycle [53]. The amplified region usually contains the *RUNX1* gene, so FISH is used to detect this abnormal chromosome. Currently, iAMP21-ALL is defined as the presence of three or more extra copies of *RUNX1* on a single abnormal chromosome 21 (a total of five or more RUNX1 signals per cell) [51]. This subtype of leukemia is associated with a worse prognosis and requires more intensive therapy because patients treated with standard therapy have a high relapse rate [54]. Genome profiling of patients with iAMP21-ALL has shown that they also have secondary genetic abnormalities that may be amenable to targeted therapy. A unique spectrum of secondary genetic abnormalities likely contributes to disease progression, which may also be used for improved diagnosis. These include a gain of chromosomes X, 10, or 14; monosomy 7/deletion of 7q; deletions of 11q, including the *ATM* and *KMT2A* genes; and deletions of *ETV6* and *RB1*. More than 60% of iAMP21-ALL patients had a mutation of genes related to the Ras signaling pathway, and 20% of patients had a *P2RY8*::*CRLF2* gene fusion [55]. It is also important to mention that patients with Down’s syndrome have a 10–12 times higher risk of developing acute leukemia compared to children without the syndrome [56]. Also at risk are carriers of the Robertson translocation between chromosomes 15 and 21, rob(15;21)(q10;q10)c. They are approximately 2700 times more likely to develop ALL and iAMP21. The dicentric nature of the Robertson and ring chromosome may be responsible for the iAMP21 chromosome structure [57].

### 2.2. Chromosome Rearrangements

#### 2.2.1. BCR-ABL1 (Ph+) ALL and Ph-like ALL

As a result of a translocation t(9;22)(q34;q11) between the *ABL* gene on chromosome 9 (region q34) and the *BCR* gene on chromosome 22 (region q11), the Philadelphia chromosome is formed with a *BCR*::*ABL1* tyrosine kinase fusion gene [58]. This translocation accounts for 3–4% of ALL cases in children. Its frequency increases with age; therefore, the presence of the *BCR*::*ABL1* fusion gene is more often observed in teenagers than in younger children [59]. BCR-ABL1 chimeric proteins may differ in molecular weight depending on the site of *BCR* gene disruption. In children, there is a shorter form with a mass of 190 kD, which is characterized by worse treatment outcomes and a 5-year OS < 10% [60]. The most common co-occurring genetic abnormalities in Ph + ALL patients are deletions of the *IKZF1*, *PAX5*, and *EBF1* genes. These alterations can be found in 80%, 50%, and 14% of patients with Ph + ALL, respectively [61,62,63]. Deletion of *CDKN2A/2B* also occurs in this group of patients, with a frequency of approximately 50% [64].

Prior to the introduction of therapy with tyrosine kinase inhibitors (TKIs), pediatric patients with Ph + ALL were treated with intensive chemotherapy followed by HSCT at the first complete remission in a patient. Generally, they had a very low survival rate. The study results showed poor median disease-free survival and overall survival despite intensive consolidation. The International Ponte di Legno Childhood ALL Consortium reported that between 1985 and 1996, 326 patients diagnosed with Ph + ALL had 7-year EFS and OS rates of 25% and 36%, respectively [65,66]. For a long time, HSCT in first remission was the only chance for improvement. This method of treatment, however, had drawbacks in terms of toxicity in long-term use [67]. The use of TKIs in combination with chemotherapy completely changed the fate of patients with Ph + ALL. New treatment regimens have led to an increase in 5-year disease-free survival to 70% and CHR rates between 90 and 100% [68,69]. In the case of prognosis of patients with Ph + ALL and secondary rearrangements, *IKAROS* deletion is associated with shorter disease-free survival and shorter cumulative incidence of relapse [70]. In one study, it was observed that the loss of *CDKN2A/B* was also associated with worse patient outcomes compared to patients without this deletion [64]. Additionally, a study by Mullighan et al. suggests that *CDKN2A/2B* deletions may contribute to drug resistance [71]. Studies by the Children’s Oncology Group, as well as the multinational European Ph + ALL intergroup study (EsPhALL), have shown that prolonged administration of imatinib in combination with appropriately selected chemotherapy significantly prolongs EFS and OS compared with the control group. It is also significant that HSCT consolidation in CR1 did not provide better survival compared with chemotherapy and TKIs alone [72,73]. Mutations in the kinase domain of *ABL1* (most frequently T315I) induce TKI resistance and are observed in patients treated with TKI monotherapy and less common in children treated with intensive chemotherapy [74]. Dasatinib or ABL second-generation TKI used in combination with COG-based chemotherapy or EsPhALL is also an effective targeted treatment strategy in pediatric Ph + ALL [75]. Current treatment approaches to mitigate the poor outcome of BCR-ABL1 ALL include frontline treatment with the third-generation TKI ponatinib with chemotherapy [76].

Ph-like or BCR-ABL1-like ALL occurs with a frequency of about 15% in children, 21% in adolescents, and 20–24% in older adults with BCP-ALL [77]. This subtype of ALL was first described in 2009 by Mulligan and by den Boer [78,79] and it shows a similar gene expression profile to BCR-ABL1 ALL, despite the lack of *BCR*::*ABL1* fusion [80]. Similar to Ph + ALL, a hallmark of Ph-like ALL is the high frequency of *IKZF1* alterations (70% to 80%) that acquire stem-cell properties, result in aberrant leukemic cell adhesion, and induce TKI resistance. Several types of *IKZF1* abnormalities have been observed in Ph-like B-ALL including deletion of the entire locus, subgroups of exons, or of genes upstream [81]. Another gene that is altered in approximately 30% of patients with Ph-like B-ALL is *PAX5*. *IKZF1* and *PAX5* alterations often occur together [82]. Among Ph-like B-ALL, *CRLF2* rearrangements are equally common and consist of a translocation of the immunoglobulin heavy chain gene *IGH* to *CRLF2* (*IgH*::*CRLF2*) or fusion due to an interstitial deletion of the PAR1 region centromeric to *CRLF2* in chromosomes X and Y (*P2RY8*::*CRLF2*) [83]. The frequency of these alterations is 24% in children with standard-risk NCI disease to 60% in adolescents [84,85]. More than 90% of Ph-like ALL cases have a large number of genetic changes that activate genes for cytokine receptors and kinase signaling pathways. These include alterations in JAK-STAT pathway genes (involving *CRLF2*, *JAK2, EPOR*, and other genes in this pathway, namely *TYT2*, *IL7R*, *SH2B*, *JAK1*, *JAK3*, *TYK2*, *IL2RB*), ABL-class rearrangements (*ABL1*, *ABL2*, *CSF1R*, *PDGFRA*, and *PDGFRB*), Ras pathway mutations (*KRAS*, *NRAS*, *NF1*, *PTPN11*), and rare fusions (*NTRK3*, *PTK2B*, *BLNK*, *FGFR1*) [86,87]. Mentioned alterations are presented in Table 1.

Patients with Ph-like B-ALL are characterized by a higher risk of induction failure, MRD positivity, higher relapse rates (up to 70% at 3 years), and lower overall survival. CRLF2 overexpression due to rearrangements is associated with poor outcomes with 4-year relapse-free survival of 35% with *CRLF2* compared to 71% without *CRLF2* alterations [88,89,90]. Studies also show that CRLF2 overexpression with co-occurring *IKZF1* deletion is associated with an increased risk of relapse even with low MRD levels [91,92,93].

The optimal treatment for pediatric ALL similar to BCR-ABL1 has still not been established. The heterogeneous genomic landscape and the diverse set of targetable kinase-activating changes of BCR-ABL1-like ALL require precise therapeutic management because conventional chemotherapy usually gives poor results. Patients with ABL rearrangements are most commonly treated with the ABL1 inhibitor imatinib and the dual ABL1/SRC inhibitor dasatinib [58,94,95]. Studies show that patients with variant *ATF7IP*::*PDGFRB* fusion, which is characterized by unfavorable outcomes, can be successfully treated with dasatinib [96,97]. These results of Mullighan’s study demonstrate that JAK kinase mutations are not limited to patients with DS-ALL but also occur in about 10% of high-risk pediatric B-progenitor ALL patients. The majority of the identified *JAK* mutations occurs in the pseudokinase domain of JAK2 in a region (R683) distinct from the predominant mutation (V617F) seen in polycythemia vera and related myeloproliferative diseases [98]. The JAK1/JAK2 inhibitor ruxolitinib is considered the most effective strategy for JAK-STAT activating mutations [95]. JAK2 inhibitors can also be used to treat patients with *CRLF2* rearrangements [99]. Three other clinical trials have also investigated ruxolitinib in combination with chemotherapy for the treatment of high-risk ALL (NCT03117751, NCT03571321, and NCT02420717). Furthermore, ruxolitinib therapy was well tolerated and induced morphologic remission. These early findings suggest with JAK inhibitors in combination with chemotherapy may improve outcomes for patients with this high-risk ALL subtype [100].

It has also been observed that patients with rare fusions, such as *BLNK*, *NTRK3*, and *TYK2*, can be treated with TRK-targeted inhibitors such as entrectinib and larotrectinib [101,102]. Immunotherapy with blinatumomab, inotuzumab, and CAR-T cells is also becoming increasingly important [103,104]. Clinical trials from the HOVON research group indicate that allogeneic stem cell transplantation (ASCT) in first complete remission can improve outcomes in Ph-like B-ALL. During the study, only one relapse was observed among the five patients who underwent ASCT, while 9 of the 15 patients treated with chemotherapy alone relapsed. However, it should be noted that this study was conducted on a limited number of patients [79]. A study by Moujalled et al. examined the effectiveness of combination therapy consisting of BCL-2 and MCL-1 inhibitors in preclinical models of Ph-like ALL. During the study, it was observed that the use of venetoclax and MCL-1 (S63845) in Ph-like cell lines increased the efficacy of dexamethasone. The addition of these drugs significantly potentiated the moderate killing efficacy observed with BCL-2 or MCL-1 inhibitors alone. Equivalent combinations of BCL-2 and MCL-1 inhibitors showed potent killing (50% lethal concentration (LC50) < 100 nM) in Ph-like cell lines. Regardless of the combination, each BH3-mimetic pair showed better synergy than the combination of each BH3-mimetic with TKIs or steroids [105]. Study of patients with Ph-like ALL and ABL class kinase rearrangements demonstrated the efficacy of TKI use during first-line treatment or at relapse. In a group of 24 patients with Ph-like ALL, 12 cases had an *ABL1* fusion, and 9 cases had a *PDGFRB* rearrangement. In addition, there were single cases of *ZC3HAV1*::*ABL2*, *MEF2D*::*CSF1R*, and *ZMYM2*::*FGFR1*. The identification of ABL class fusions allowed early initiation of TKI therapy, resulting in a 3-year EFS of 55% and OS of 77%. The results of this study showed that prospective screening strategies should be generalized to identify high-risk patients and allow earlier implementation of TKI-based intervention [106].

**Table 1:** The frequency and spectrum of genetic alterations in Ph-like ALL.

Genetic Alteration Class	Frequency of Occurring	Genes Involved	Targeted Therapy	References
JAK-STAT signaling rearrangements	40%	*CRLF2*	JAK2 inhibitor	[58,77,102]
		*JAK2*		
		*EPOR*		
		*TSLP*		
		*IL2RB*	JAK1/JAK3 inhibitor	
		*TYK2*	TYK2 inhibitor	
ABL-class fusions	10–15%	*ABL1*, *ABL2*, *PDGFRA*, *PDGFRB*, *CSFIR*, *LYN*	Imatinib/dasatinib	[58,99]
RAS pathway mutations	4%	*KRAS*, *NRAS*, *PTNP11*, *CBL1*, *NF1*, *BRAF*	MEK inhibitors	[58,102]
Rare subtypes	1%	*NTRK3*	Crizotinib	[58,77,95,99,102]
		*BLNK*	SYK/MEK inhibitors	
		*FGFR1*	Dasatinib/sorafenib	
		*PT2KB*	FAK inhibitors	
		*FLT3*	FLT3 inhibitors	
		*DGKH*	-	

#### 2.2.2. KMT2A Rearrangements

The histone lysine [K]-Methyl Transferase 2A gene (*KMT2A*), which was formerly known as the mixed-lineage leukemia (*MLL*) gene, is located on chromosome 11q23 [107]. It is present in 5% of children and 10% of adults with ALL. However, when it comes to leukemias among infants, the incidence of *KMT2A* rearrangements is 70–80%, and they are frequently acquired in utero [108,109,110]. The *KMT2A*-rearranged ALL is associated with the precursor B-cell ALL immunophenotype with the expression of CD19 antigen, lack of CD10, and also co-expression of myeloid markers such as CD15, CD33, and CD68 [111,112]. It presents both lymphoid and myeloid features with bimodal incidence. The first peak occurs postnatally in the first 2 years with a decline during the pediatric and young adult phase, until it increases again with age. However, this biphasic distribution in age is not well understood yet [113].

Leukemia-associated translocations involving 11q23 lead to fusions of *KMT2A* to more than 90 different partner genes [113,114]. *AFF1* (formerly named *AF4*) was reported to be the most frequent partner gene with an especially poor prognosis for *KMT2A*::*AFF1* t(4;11)(q21;q23) fusion. Its prevalence is estimated at 50% in infants with KMT2A-rearranged ALL [113,115]. The second most common fusion is *KMT2A*::*MLLT3* (previously *KMT2A*::*AF9*) from t(9;11)(p22;q23), while the third is *KMT2A*::*MLLT1* (previously *KMT2A*::*ENL*) that originated from t(11;19)(q23;p13.3) translocation [116]. Additionally, it has been shown that patients with a *KMT2A* breakpoint in intron 11 presented poorer outcomes [117]. Other main partner genes in infant ALL patients involve *ENL*, *MLLT10* (formerly *AF10*), and *MLLT4* (formerly *AF6*) [113]. Recently, novel rearrangements in acute leukemias such as *KMT2A*::*BTK* with (X;11)(q22.1;q23.3), *KMT2A*::*NUTM2A* with t(10;11)(q22;q23.3), and also *KMT2A*::*PRPF19* with inv(11)(q12.2;q23.3) were detected. However, they were most predominant in acute myeloid leukemia (AML) or T-cell ALL [118].

Prognosis in the group of infants with ALL and 11q23 alterations is particularly poor compared to other children with diagnosed ALL [119]. Additionally, due to similar outcomes in the adult population, it may be associated with a poor prognosis in all ages [120]. Interestingly, particularly in infants, *KMT2A*-r acute leukemias are more likely to proceed with hyperleukocytosis and central nervous system disease (more than five leukemic cells/μL found in cerebrospinal fluid) involvement [121].

Rarely, there is also a “therapy-related leukemia” that occurs after exposure to topoisomerase II inhibitors such as etoposide or doxorubicin. The incidence of the 11q23 rearrangements in these cases is 70–90%, which includes translocations that are predominant in children [114,122].

Based on whole-genome sequencing (WGS) and whole-exome sequencing studies, it was suggested that *KMT2A* rearrangement alone may be sufficient for inducing leukemia in some cases due to the harboring of very few cooperating lesions [123]. Despite the low overall number of cooperating mutations among patients with infant ALL, the high frequency of mutations in the tyrosine kinase/PI3K/RAS signaling pathways were identified, including recurrent ones in *KRAS* and *NRAS*, and also non-recurrent mutations in *FLT3*, *NF1*, *PTPN11*, and *PIK3R1* [123,124]. Additionally, a mouse model of *KMT2A*-rearranged leukemia proved that subclonal *FLT3* mutations accelerate disease, with its most frequent mutation providing stimulatory factors [125]. Interestingly, mouse genetic models showed that Dot1L (Disruptor of Telomeric-Silencing 1 Like) plays an important role in *KMT2A*-rearranged ALL initiation and maintenance. Possibly, the expression of *KMT2A*-translocation partners, including *AF9*, *ENL*, and *AF10*, depends on recruiting excessive DOT1L activity to their target loci. Therefore, the use of Dot1L inhibitors may constitute a promising approach for targeted therapy in this aggressive leukemia [126]. Additionally, there are several other possible therapeutic targets including inhibitors of bromodomain, menin, BCL-2, and also polycomb repressive complex inhibition [114,127].

#### 2.2.3. TCF Rearrangements

Transcription factor 3, also known as *TCF3* or *E2A*, is a member of the E protein (class I) family of helix–loop–helix transcription factors and plays an important role in lymphopoiesis, as it is required for proper development and differentiation of T and B lymphocytes [128]. In addition, *TCF3* regulates also the development of the central nervous system. E proteins are involved in initiating transcription and binding to regulatory E-box sequences on target genes [129]. This gene is involved in several chromosomal translocations that are associated with lymphoid malignancies. Translocation t(1;19)(q23;p13.3), generating *TCF3*::*PBX1* fusion, found in pre-B cell acute lymphoblastic leukemia, results in a chimeric protein that is directly associated with malignant transformation of hematopoietic cells and is observed in both adults and children with B-ALL at an overall frequency of 5–6% [82,130].

Several studies have identified overexpression of ROR1 and Wnt16b in t(1;19) BCP-ALL cells compared to other BCP-ALL subtypes, suggesting a possible common signaling pathway for Wnt16b-ROR1 in these cells [131,132]. High expression of ROR1 in malignant B cells is associated with activation of the noncanonical Wnt pathway through autocrine or paracrine binding of Wnt5a to modulate cell proliferation, chemotaxis, and survival [133,134]. Overexpression of ROR1 in *TCF3*::*PBX1* cells may be a promising possibility for a targeted treatment strategy to reduce the cytotoxic effects on healthy B lymphocytes [135].

For many years, ALL patients who have this translocation were thought to have a poor prognosis, mainly due to higher CNS involvement and relapse [136]. Recent studies have shown that intensive multi-component chemotherapy results in better outcomes (OS > 80%); therefore, this subtype of leukemia is now classified as favorable or intermediate [137,138]. The high similarities between the conditional *TCF3*::*PBX1* Tg mouse model and human *TCF3*::*PBX1* + ALL provide an opportunity to develop potential new treatment therapies. *TCF3*::*PBX1* + mouse leukemias show consistent loss of tumor-suppressor genes (*PAX5* and *CDKN2A/2B*) and activation of signaling pathways by point mutations (JAK/STAT, RAS/MAPK). These observations suggest the possible efficacy of targeted therapy in pre-BCR signaling by dasatinib and the JAK/STAT pathway by ruxolitinib [139]. The studies also point to the effectiveness of ponatinib, but not imatinib [135].

Another *TCF3*-associated translocation, t(17;19)(q22;p13), initiates *TCF3*::*HLF* fusion. This bond includes the transactivation *E2A* domains and the basic leucine zipper (bZIP) DNA-binding HLF domain [140]. *TCF3*::*HLF* leukemia is a rare subtype of B-cell acute lymphoblastic leukemia (B-ALL) with extremely poor prognosis, which accounts for less than 1% of childhood B-ALL. It is clinically manifested by hypercalcemia and disseminated intravascular coagulopathy [141,142]. *TCF3*::*HLF* expression induces transcriptional alterations in pre-leukemia cells. By its activity, this gene manipulates the transcription factor *LMO2* as well as the transcriptional repressor SNAI1 (SLUG), which is responsible for embryonic development and cell apoptosis [143,144]. The *TCF3*::*HLF* corresponds to stem cell and bone marrow gene features and is characterized by *PAX5* and *VPREB1* deletions and aberrations in the Ras pathway genes. Moreover, it has a significant impact on recruiting the *EP300* gene to enhance MYC and EP300 inhibition, which reduces the ALL increase [20,21]. Despite intensive treatment and transplantation of hematopoietic stem cells (HSCT), it has a high failure rate [89,90]. The peak incidence is at 15 years of age and is usually manifested by relapse and death within two years of diagnosis [20,145]. The poor prognosis and small number of described cases result in the lack of a specific chemotherapy protocol for these patients. However, recent studies have highlighted venetoclax, a BCL20 inhibitor, as a potential therapeutic factor [145]. In addition, CAR-T cell therapy appears to be a promising therapeutic element to improve remission in patients with *TCF3*::*HLF* ALL [146].

#### 2.2.4. ETV6::RUNX1-Rearrangements and ETV6::RUNX1-like ALL

Fusion of the *ETS* variant 6 (*ETV6*) and Runt-related transcription factor 1 (*RUNX1*) genes arises from the t(12;21)(p13;q22) translocation [147]. *ETV6*::*RUNX1* fusion, which is also known as *TEL*::*AML1*, affects approximately 25% of children with precursor-B phenotype ALL and is considered the most common genetic alteration among these patients [148]. *ETV6-RUNX1*-positive ALL, first reported by Romana et al. in 1994, is thought to arise prenatally and may be preceded by a pre-leukemic phase [149]. Additionally, it is present in less than 5% of adolescents and young adults (AYA) and adult patients [148]. ETV6, which is also known as TEL, belongs to the ETS protein family. It is encoded by the *ETV6* gene at chromosome 12p13 and plays an important role as a transcriptional repressor [150,151]. RUNX1 is a DNA-binding protein encoded by a gene located on the 21q22 chromosome. It is homologous to the Drosophila pair-rule gene runt and was also proven to act as a transcriptional organizer and to regulate the expression of different hematopoietic specific genes [152,153]. Due to their ability to encode transcription factors, both *ETV6* and *RUNX1* play a crucial role in hematopoiesis [150,153].

The presence of *ETV6*::*RUNX1* fusion in cord blood samples suggested that this disease may have originated prenatally [149,154]. However, fusion alone is not likely to be responsible for causing overt leukemia, as 5% of healthy newborns are diagnosed with this alteration at birth [155]. Thus, *ETV6*::*RUNX1* is considered as a first hit in the leukemogenesis process. Therefore, inducing complete leukemic transformation requires prolonged latency with secondary genetic aberrations. These second acquired hits include different alterations in genes that are associated with B-cell maturation, including *ETV6*, *PAX5*, *ATF7IP*, and others [156,157,158]. *ETV6*::*RUNX1* fusion is frequently accompanied by 12p deletion in the region that contains a non-translocated allele of *ETV6* [159,160]. Other genetic changes such as *KMT2A* aberrations, deletions of 6q and 9p (including the *CDKN2A* gene) numerous trisomies (chromosomes 21, 4, 10, 16), an extra copy of *RUNX1*, and duplication of the derivative chromosome 21 were associated with poorer outcomes, especially in relapsed patients [161,162,163]. Interestingly, Loncarevic et al. observed that duplication of the normal chromosome 21 resulted in an extra *RUNX1* allele in the case of trisomy 21. This duplication was present in 78% of cases of relapse patients and also in 15% of patients at initial diagnosis [164]. Furthermore, another chromosomal aberration such as 9p deletion associated with *MTAP*, *CDKN2A*, *CDKN2B*, *DMRTA1*, and *FLJ35282* gene loss was indicated [165]. In addition, it was suggested that environmental factors, such as infection, may also be linked with B-ALL development [166].

*ETV6*::*RUNX1* fusion, which belongs to the favorable-risk genetics (FRG) group, is also characterized by excellent outcomes [167]. On the other hand, the NOPHO-ALL-1992 protocol showed that *ETV6*::*RUNX1*-rearranged childhood ALL was associated with common late relapses and a greater male incidence ratio. However, the OS was good with 94% at 5 years and 88% at 10 years. Interestingly, second or later remission treatment was efficient [168]. FRALLE 93 protocol also underlined the importance of late relapses that occurred with a frequency of 20% in the same patient group as mentioned above. The second complete remission was higher compared to previous studies, with an efficacy of 98% [169].

Kato’s study showed excellent outcomes with the use of short maintenance therapy in the *ETV6*::*RUNX1* cytogenic group. The disease-free survival reached 93.8 ± 6.1% [170]. JACLS ALL-02 protocol treatment appeared to be also successful with similar outcomes as in western countries [171]. In addition, it has been shown that a group of *ETV6*::*RUNX1*-rearranged B-cell precursor ALL (BCP-ALL) patients may benefit from reductions in the intensity of chemotherapy. The favorable outcomes were also highlighted in cases of good initial MRD responses and reduction treatment [171,172,173]. The use of an improved Berlin–Frankfurt–Münster (BFM) protocol that employed the intensive L-asparaginase and high-dose methotrexate may be also beneficial to patients with the *ETV6*::*RUNX1* fusion transcript [174]. What is worth mentioning is that previous studies confirmed that observation by demonstrating *ETV6*::*RUNX1*-positive lymphoblast sensitivity to high-dose methotrexate and L-asparaginase in vitro [168]. Wang’s study highlighted that a longer treatment course may be considered as one of the most important factors in determining prognosis due to the majority of relapses occurring after the therapy [168,174].

Recently, a new subtype, *ETV6*::*RUNX1*-like ALL, was reported in Lilljebjörn et al.’s study. Interestingly, despite the lack of *ETV6*::*RUNX1* fusion, it has similar gene expression profiles and immunophenotype (CD27 positive, CD44 low to negative) to *ETV6*::*RUNX1* ALL [175,176]. *ETV6*::*RUNX1*-like accounts for approximately 2–3% of children patients with BCP-ALL [177]. Additionally, more than 80% of *ETV6*::*RUNX1*-like subtype cases occur in children [130,178]. This particular subtype is enriched with ETV6 fusions including *IKZF1*::*ETV6* and *ETV6*::*ELMO1*; other chromosomal rearrangements such as *TCF3*::*FLI1*, *FUS*::*ERG*, and *IKZF1*; and additionally *ARPP21* deletions [130,175,176,179]. This picture suggests that *ETV6::RUNX1*-like ALL is characterized by global deregulation of lymphoid development [130]. Previous studies showed that the *ETV6*::*RUNX1*-like subtype had a relatively favorable outcome with very few reported relapses [130,179]. However, recent studies proved that it had poorer outcomes compared to *ETV6*::*RUNX1* [180]. Additionally, among the 16 B-ALL subtypes, *ETV6*::*RUNX1*-like patients had the worst five-year EFS rates along with *KMT2A*-rearranged and MEF2D-rearranged ALL patients. The average result of 5-year EFS was 66.7%. However due to the small number of patients with *ETV6*::*RUNX1*-like ALL (9), more studies are required in this field [181]. It is possible that an *ETV6*::*RUNX1*-like subtype may benefit from higher-intensity therapy [180].

### 2.3. Other Rearrangements

#### 2.3.1. IKZF1

The *IKZF* genes encode transcription factors belonging to the zinc finger DNA-binding proteins group. Due to N-terminal zinc finger domains, these proteins mediate direct interactions with DNA. The IKZF family is composed of five subtypes: IKAROS (encoded by the gene *IKZF1*), HELIOS (*IKZF2*), AIOLOS (*IKZF3*), EOS (*IKZF4*), and PEGASUS (*IKZF5*) [182].

*IKZF1* is located on the short arm of chromosome 7 in position 7p12.2, and the biology of *IKZF1* is complex because this gene consists of 8 exons and encodes 11 different splice variants [183]. IKAROS, as well as other members of this family, is expressed in lymphocytes. It is crucial for the regulation of lymphocyte-specific genes [184]. It plays a key role in hematopoiesis, differentiation, and proliferation of all lymphoid lineages, especially in the activation and development of B cells [185,186]. It also helps regulate genes that control cell cycle progression and cell survival [183,187,188,189]. Furthermore, IKAROS has also been shown to regulate the function of other immune cells, i.e., natural killer (NK) cells, innate lymphoid subsets, and plasmacytoid dendritic cells [190,191]. Defects within IKAROS inhibit precursor B lymphocytes that predispose them to malignant transformation [192].

The frequency of *IKZF1* deletions in children for all B-ALL variants is estimated at 16–27% [62,81,158,193]. These deletions often involve the entire gene (DEL 1–8), resulting in loss of wild-type *IKZF1* expression, but can also occur as focal deletions (exons 4–7) [194]. Deletions of the whole gene and partial deletions that affect at least the starting codon located in exon 2 result in haploinsufficiency. Deletions that affect the DNA-binding domain in exons 4–7 (known as isoform 6) exert a dominant–negative effect over the unaffected allele, resulting in a loss of the tumor suppressor function attributed to wild-type *IKZF1* [195]. *IKZF1* alterations are frequently observed in BCR-ABL1-positive ALL (85%) and BCR-ABL1-like ALL (70%) [196]. In terms of adults, genomic changes in *IKZF1* are found in 40% of cases, with a higher frequency in poor prognosis, including BCR-ABL1 (70%) or BCR-ABL1-like (40%) B-ALL [14,57]. The detection of *IKZF1* deletions was found to be associated with older age at diagnosis, higher levels of white blood cells, and higher levels of MRD after induction and consolidation [197]. Recently, a new subgroup characterized by the *IKZF1* missense mutation p.Asn159Tyr (N159Y) affecting the DNA-binding domain was identified through a distinct gene-expression profile. The specific gene-expression profile also differed from those BCP ALL cases with other known *IKZF1* alterations. In addition, an increasing number of cases with fusion transcripts involving *IKZF1* have been described (*IKZF1*::*PRDM16*, *IKZF1*::*NUMT1*, *IKZF1*::*ETV6*, *IKZF1*::*CDK2*, *IKZF1*::*ZEB2*, *IKZF1*::*SETD5*, *IKZF1*::*STIM2*) [178].

The presence of alterations in BCR-ABL1-positive ALL may result in resistance to tyrosine kinase inhibitor therapy and contribute to poorer treatment outcomes [198]. In contrast, *IKZF1* deletions are rarely detected in *TCF3*-rearrangements (3%) and ETV6-RUNX1-positive BCP-ALL (3%). Among other subtypes such as hyperdiploid and B-other leukemia, the frequency of these alterations ranges from 15% to 20% [196].

B-ALL patients with *IKZF1* abnormalities have a reduced 5-year EFS and overall survival, and an increased risk of relapse [158]. A Dutch study reported that the individuals treated with standard therapy had a 12-fold increased risk of relapse [199]. A study conducted on Japanese pediatric BCP-ALL patients showed a 5-year EFS of 62.7% of patients compared with an 88.8% rate in those without *IKZF1* deletion. Respectively, the OS rate was 71.8% vs. 90.2% [200]. Research in Taiwan exhibited even worse values of EFF (15% vs. 76%) and OS (38% vs. 78%) [201]. Similar values were also shown in studies conducted in Italy, Sweden, and Germany [197,202,203,204]. Recently, scientists have begun to distinguish the subtype IKZF1 plus, which has a very poor prognostic result. It is characterized by an *IKZF1* deletion that coexists with deletions in *CDKN2A*, *CDKN2B*, *PAX5,* or *PAR1* in the absence of the *ERG* deletion. The study conducted by the international multicenter trial AIEOP-BFM ALL 2000 on 991 patients showed that only ~53% of patients with the *IKZF1* plus type had a 5-year event-free survival, compared to ~79% of patients with *IKZF1* deletion alone and ~87% in patients without the *IKZF1* deletion. The 5-year cumulative relapse rates were 44 ± 6%, 11 ± 4%, and 10 ± 1%, respectively [205]. Moreover, it was shown that *IKZF1* plus had a strong prognostic effect only in patients with measurable MRD, with a leukemic cell load greater than 10^−4^ after induction treatment [197].

A growing number of studies suggests that *IKZF1* dysfunction may also lead to activation of the PI3K/AKT/mTOR pathway, and this, in turn, promotes resistance to glucocorticoids, which are essential drugs in the treatment of patients with ALL [206,207].

It has been shown that deregulation of the *ERG* transcription factor does not significantly affect the prognosis of the BCP-ALL subtype. A study carried out on a group of patients among whom approximately 40% had a deletion of the *IKZF1* gene showed that its presence does not affect the prognosis of BCP-ALL when it coexists with an *ERG* deletion. These patients had excellent treatment outcomes, exceeding 90% at five years [208,209].

In addition to their importance in leukemia pathogenesis and unfavorable prognosis in pediatric B-ALL, *IKZF1* gene alterations are valuable prognostic markers and should be included in algorithms for early risk stratification in the treatment of pediatric BCP-ALL.

#### 2.3.2. PAX5

The transcription factor paired-box domain 5 (*PAX5*) located on 9p13 chromosome is a crucial regulator of the early stages of B cell development [210,211]. It belongs to one of four groups of the highly conserved paired box (PAX) domain family that is involved in the hematopoietic system and cell differentiation [212]. At the molecular level, *PAX5* induces B-cell differentiation by activating B-cell-specific genes, which are crucial components of the pre-BCR signaling pathway [213]. Additionally, it is responsible for inhibiting progress toward other cell lineages by PD-1 and NOTCH1 transcription factors’ negative regulation and M-CSFR inhibition [63,214]. Any change in *PAX5* expression, which is limited to B-cells only, can lead to leukemogenesis and trigger malignancy [215].

**Figure 2:**
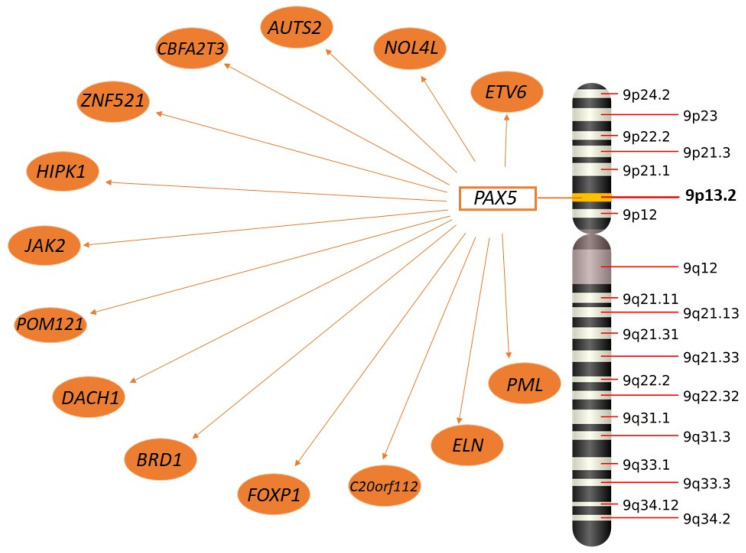
PAX5 partner genes.

The *PAX5* gene constitutes the most important target of somatic mutations in BCP-ALL in children, with its mutation being considered as one of the most common genetic lesions in B-ALL [216,217]. *PAX5* alterations include DNA copy number variations (CNVs), sequence mutations, and chromosomal translocations with an incidence of 30% in case of CNVs, 5–9% in non-silence sequence mutations, 5–7% in children, and 2–4% in adults with chromosomal translocations [130,178,218,219]. PAX chromosomal translocations with at least 24 partner genes may cause expression of chimeric in-frame fusion transcripts [130] (Figure 2).

Recent studies presented two subtypes of PAX-driven B-ALL. First *PAX5*-altered (PAX5alt) includes different alterations such as rearrangements, focal/intragenic amplifications, or mutations. Its occurrence was most common among children and the AYA population. It was present in 7–10% of cases of children with BCP-ALL [130,178]. According to National Cancer Institute criteria, children in this subtype had a bigger chance to be classified as high risk [130]. The above-mentioned alterations included most frequently *PAX5*::*ETV6*, *PAX5*::*NOL4L, PAX5*::*AUTS2,* and *PAX5*::*CBFA2T3* rearrangements; non-silent sequence mutations such as *PAX5* p.Pro32Ser (P32S), p.Pro34Leu (P34L), and p.Arg38Cys (R38C)/p.Arg38His (R38H); and most frequently focal intragenic amplification of PAX5 (PAX5amp) [130,177]. Furthermore, other PAX5 fusion genes include *HIPK1*, *POM121*, *JAK2*, *DACH1*, *BRD1*, *ELN*, *FOXP1*, *ZNF521*, *PML*, and *C20orf112* [156,211,220,221,222]. Additionally, other genetic alterations associated with cell cycle and transcriptional regulation or epigenetic modification were also identified in PAX5alt patients [130].

The second subtype of PAX-driven B-ALL is the hotspot mutation *PAX5* p.Pro80Arg (P80R) with an occurrence of 3%–4% in children patients and 4% of adults with BCP-ALL [130,178]. This mutation can be accompanied by biallelic deletion of *CDKN2A*, inactivating mutations in the epigenetic factor SETD2, and also inactivation of the wild-type PAX5 allele. The last one may be caused by deletion, loss-of-function mutation, or copy-neutral loss of heterozygosity [130,177,223,224]. Additionally, the fact that PAX P80R coexists with mutations of the signaling pathway that include *Ras*, *JAK/STAT*, *FLT3*, *BRAF*, and *PIK3CA* creates the potential for targeted therapies [130,224]. Interestingly, both PAX5alt and P80R are associated with intermediate outcomes. Because of the high heterogeneity in genetics, PAX-driven subtypes may require diverse therapy agents such as a combination of chemotherapy and multi-inhibitors, and also immunotherapy [177].

#### 2.3.3. DUX4

The B-progenitor ALL subtype is characterized by deregulation of the homeobox transcription factor gene *DUX4*, which is reported in 4–7% of childhood B-ALL cases [225]. It was indicated that the *DUX4* rearrangement is a clonal event acquired in the early stage of leukemogenesis [226]. It has been shown that CD2 and CD371 antigen expression was strongly associated with *DUX4* positivity in B-ALL. CD2 expression alone allows the detection of only part of the cases. However, it is possible to identify this subtype with the use of only one single cell surface protein, because the CD371 antigen is pathognomonic of *DUX4*-positive leukemia [227].

*DUX4* fusions may induce leukemogenesis by their reposition in the proximity of the immunoglobulin heavy chain (IGH) enhancer. *DUX4* is present in the D4Z4 repeats of the subtelomeric region of chromosome 4q or the homologous region at 10q. However, D4Z4 repeats go through the process of insertion to the IGH locus on chromosome 14 [225,228]. This rearrangement leads to disruption of the highly conserved C terminus of *DUX4*, which is necessary for the process of *DUX4* oncogenic activation. As a result, the C-terminal truncated protein is expressed. A transplantation assay in mice confirmed that expression of *DUX4*::*IGH* in pro-B cells was able to generate a B cell leukemia in vivo [228].

Deregulation of ETS transcription factor gene *ERG* occurs in 5–10% of *DUX4*-rearranged leukemia cases. There is a slight peak of its incidence among AYA [20]. Studies suggested that overexpression of *DUX4* caused transcriptional deregulation of *ERG* by binding to its alternative transcription initiation site in intron 6 and also causing expression of multiple aberrant coding and non-coding *ERG* isoforms. ERGalt is one of these isoforms, which by inhibiting the wild-type ERG function, may directly contribute to leukemogenesis [227]. Mostly polyclonal *ERG* deletions, which occur with a frequency of 3–7% in the pediatric BCP ALL population, are a secondary event that is present only in a subset of *DUX4*-positive cases. Interestingly, ERGdel presence was associated with a positive prognosis in patients with *IKZF1*-deletion [229]. Considering the presence of *ERG* deletion only in some of the cases, there was a strong need to find a highly specific surrogate marker such as above mentioned CD371 in order to identify or determine the prognostic relevance of DUX4 positivity in this leukemia subtype [226,227].

Recent studies suggested that *DUX4*-rearranged leukemia was associated with 93% of 5-year EFS and OS in pediatric patients [130]. Additionally, AYA patients presented longer disease-free survival after complete remission (CR) [229]. In the context of applied intensive chemotherapy, it appears that *DUX4*-r in B-ALL patients presents favorable outcomes [225]. The differences in prognosis between pediatric and adult patients were highlighted with better outcomes in the first group of patients [130,219]. Furthermore, the presence of concomitant genetic alterations in *DUX4*::*ERG* ALL did not affect its favorable outcomes [226]. Due to positive effects in reducing cell proliferation by targeting the DUX4 fusion transcript by utilizing shRNA, it is possible that DUX-rearranged B-ALL may be prone to targeted therapy [228].

#### 2.3.4. ZNF384

The zinc finger protein 384 (*ZNF384*) gene located within chromosome 12p13.31 is responsible for coding a putative C2H2 zinc finger transcription factor that is involved in the regulation of matrix metalloproteinases [230,231]. The *ZNF384* rearrangements account for approximately 3–5% of childhood cases, 7–10% of AYA, and 3–8% of adult patients with BCP-ALL. They are also associated with intermediate outcomes [177,219]. These alterations may be manifested most frequently as classical pre-B ALL without lineage aberrancy, but also as B-ALL with the expression of cell surface markers of myeloid lineage (CD13/33), or B/myeloid mixed phenotype acute leukemia (MPAL) [232]. Leukemic cells from patients with *ZNF384* fusions were reported to present CD10-negative or CD10 low immunophenotypes [219,233]. In addition, studies showed that *ZNF384* rearrangements are acquired in a hematopoietic stem cell that is primed for lineage aberrancy [94].

Several 5′ fusion partners for *ZNF384* rearrangements were identified, including most commonly *EP300*, *TCF3*, and *TAF15* and also *ARIDIB*, *BMP2K*, *CLTC*, *CREBBP*, *EP300*, *EWSR1*, *NIPBL*, *SMARCA2*, and *SYNRG* [219,234,235]. Fusions such as *EP300*::*ZNF384* and *TCF3*::*ZNF384* have already been clinically characterized. The Hirabayashi et al. study pointed out that rearrangements with *EP300* and *TCF3* partner genes occurred with frequencies of 43% and 31%, respectively. The median age for patients with *EP300*::*ZNF384* was 11 years, while for *TCF3*::*ZNF384* it was 5 years. The *EP300*::*ZNF384* outcome was excellent with a lower cumulative relapse rate compared to *TCF3*::*ZNF384*, with frequently observed late relapses [236].

Deletions in lymphoid regulator genes including *LEF1*, *EBF1*, *CDKN2A*, *FBXW7*, and *ETV6* have also been detected in ZNF384 rearranged ALL [233]. In the case of rearranged BCP-ALL, cardiotrophin-like cytokine factor 1 (CLCF1) is upregulated, which by binding to CRLF1 results in activating the JAK-STAT signaling pathway and B-cell proliferation in vivo. Additionally, the MAPK signaling pathway is also significantly upregulated in this subtype [178,237]. In 60% of the patients with *ZNF384* fusions, alterations in their signaling molecules, such as *NRAS* and *FLT3*, were observed [177]. Due to *FLT3* overexpression and responsiveness to *FLT3* inhibition in this ALL subtype, targeted therapy with the multi-kinase inhibitor sunitinib should be considered [238].

#### 2.3.5. CRLF2 Deregulation

The cytokine receptor-like factor 2 (*CRLF2*) gene encodes a member of the type I cytokine receptor family. The encoded protein is a functional receptor for thymic stromal lymphopoietin (TSLP). Together with the interleukin 7 receptor (IL7R) and TSLP, the encoded protein forms a signaling complex that controls processes such as cell proliferation and B-cell development through activation of STAT3, STAT5, and JAK2 pathways [239,240].

*CRLF2* rearrangements occur in approximately 5% of patients with BCP-ALL. This frequency is higher in the B-other ALL subtype (30%) and in patients with Down syndrome (>50%) [81,241]. *CRLF2* genetic aberrations are mainly the result of *P2RY8*::*CRLF2* fusion caused by intrachromosomal deletions within the pseudoautosomal region (PAR1) located in the p arm of the sex chromosomes [242]. *P2RY8*::*CRLF2* is often a secondary lesion in leukemias with iAMP21, hyperdiploidy, or dic(9;20) [243]. CRLF2 overexpression can also be caused by a translocation involving the immunoglobulin heavy chain (IGH) locus on chromosome 14q32.3 [244]. An important study in the aspect of *CRLF2* rearrangement is one conducted by Harvey et al. His results showed a higher frequency of these alterations in the Spanish/Latino population and a frequent coexistence of *JAK* mutations and *IKZF1* deletions [245]. *CRLF2*-rearranged ALL commonly has concomitant alterations that facilitate JAK-STAT signaling pathway activation, including sequence mutations of Janus kinases (most commonly at R683 of the pseudokinase domain of JAK2), IL-7RA, and deletions of negative regulators of JAK-STAT signaling (*SH2B3* and *USP9X*) [246].

The poor prognosis of patients with *P2RY8*::*CRLF2* is mainly caused by the high frequency of relapses [247]. This rearrangement was shown to be associated with a high relapse incidence in children treated according to the ALL-Berlin–Frankfurt–Münster protocol [248]. A study by Russell et al. showed that patients with ALL and IGH translocation had inferior overall survival compared with patients without this translocation [249]. The Harvey et al. study previously mentioned showed that in patients with disrupted *CRLF2*, the predicted relapse-free survival (RFS) at 4 years was 35.3 ± 9.5% in contrast to 71.3 ± 3.6% in the case of patients without this change [245].

Children with ALL and *CRLF2* rearrangements respond poorly to current chemotherapy. In this group of patients, a high rate of minimal residual disease was observed at the end of induction chemotherapy [250]. Therefore, new and more effective treatment regimens are still being sought. Cytometric tests performed on samples from patients with *CRLF2*-rearranged ALL showed TSLP-induced abnormal signal transduction in JAK/STAT and PI3K/mTOR pathways and the possibility of inhibition of these signaling pathways with targeted STIs. JAK inhibition resulted in inhibition of both pathways, indicating a potentially effective role for these agents in clinical practice [251].

#### 2.3.6. MEF2D Rearrangements

MEF2D is a transcription factor belonging to the *MEF2* gene family that is involved in muscle and nerve cell differentiation, blood vessel formation, and growth factor responsiveness [252]. *MEF2D* rearrangements occur in 4% of pediatric patients with B-ALL [181].

ALL patients with the *MEF2D* fusion gene have an immunophenotype with low or no CD10 expression and high CD38 expression [253]. Additional genetic alterations observed in cases with *MEF2D* rearrangement included deletions in *IKZF1* and a significantly higher prevalence of *CDKN2A/CDKN2B* deletions. Patients usually have an older age of onset and an increased number of white blood cells; therefore, they are classified as intermediate or high risk [254]. B-ALL with the *MEF2D* rearrangement also shows high levels of minimal residual disease and low event-free survival [181].

Overexpression of histone deacetylase 9 (HDAC9) can also be observed in *MEF2D*-r patients, which confers therapeutic sensitivity to histone deacetylase inhibitors such as panobinostat [253,254]. Studies suggest that staurosporine and venetoclax, which activate caspase-dependent proteolysis of MEF2D fusion proteins and apoptosis in *MEF2D* fusion+ ALL cells, may be effective in the treatment of ALL with *MEF2D* rearrangements [255].

#### 2.3.7. NUTM1

NUT midline carcinoma family member 1 (*NUTM1*), also called nuclear protein in the testis, is a chromatin regulator responsible for recruiting *EP300*, which leads to increased local histone acetylation. It is located at chromosome 15q14 [177,256]. *NUTM1* rearranged BCP-ALL is a rare subtype observed more frequently in infants than in children, with no NUTM1 fusions reported in adults. Its frequency among children with BCP-ALL is 1% [130,178,257]. Interestingly, the *NUMT1* subtype may be more prevalent in infants without *KMT2A* rearrangement [123,258]. More importantly, the outcome of *NUTM1*-rearranged ALL among infants and children is excellent [257].

The above-mentioned *EP300* stimulation by fusion proteins leads to the upregulation of the proto-oncogene BMI1 and other 10p12.31–12.2 genes in BCP-ALL [258]. Additionally, *NUTM1* fusions are associated with *HOXA* gene cluster upregulation [178]. In previous studies, common partner *BRD9*::*NUTM1* was indicated in BCP-ALL, while *BRD4*::*NUTM1* was reported in nut midline carcinoma [259]. Boer’s study suggested that within the NUTM1 subtype there are two biological subgroups. The first subgroup of HOXA9-positive NUTM1 involves a limited number of partners including *ACIN1*, *CUX1*, *BRD*9, and *AFF1*, which are specific to infants less than 9 months in age. The second subgroup of *HOXA9*-negative *NUTM1* is prevalent among infants close to 1 year old. Additionally, it increases to almost half of *NUTM1*-rearranged pediatric cases. Yet, new *NUTM1* partners are still being discovered [257].

#### 2.3.8. CDKN2A

The *CDKN2A* gene, also known as *INK4A* or *P16-INK4A*, is a cyclin-dependent kinase inhibitor gene that codes for two proteins, namely p16 (p16INK4a) and p14arf. It is located on chromosome 9 in the 9p21.3 cytogenetic band [260]. The p16 protein is a selective inhibitor of cyclin D-dependent kinases CDK4 and CDK6. The p14arf protein activates TP53 by binding directly to the p53-stabilizing protein MDM2 [261].

The most common changes in the C*DKN2A* gene are deletions. They can be found in approximately 25% of ALL patients, including 5–20% with the B-cell precursor [262]. They often co-occur with *PAX5* deletions due to recurrent 9p losses [263]. They can also be found in Ph + ALL and Ph-like ALL, and less frequently in the *ETV6*::*RUNX1* and hyperdiploid ALL subtypes [58,263,264]. In BCP-ALL, heterozygous and homozygous *CDKN2A* deletions appear to occur at approximately the same frequency. However, some studies have relied on deletion detection based on methods such as polymerase chain reaction (PCR) and immunocytochemistry, which are unable to detect heterozygous deletion. Some studies have shown that both biallelic and monoallelic mutations affect patient prognosis. However, some research suggests that only homozygous deletions have a clinical impact on patients’ surveillance [262,265]. A metanalysis by Zhang et al. showed that the presence of *CDKN2A/2B* deletion was associated with adverse OS and EFS outcomes in both pediatric and adult ALL patients [266]. The hypermethylation of the promoter of the *CDKN2A* genes has also been described in ALL patients. The prevalence of these alterations ranges from 0 to 40% in the pediatric population. Very few studies have analyzed the effect of methylation status in the CDKN2A/B promoters, most of which have not shown a significant effect on BCP-ALL progression [267].

Disruption of INK4 protein regulatory function can result in increased CDK4/CDK6 activity causing uncontrolled proliferation. Clinical trials are currently underway for pharmacological CDK4/CDK6 inhibitors such as palbociclib, ribociclib, and abemaciclib, which block the cell cycle in the G1 phase and may prevent leukemia progression [268,269,270].

#### 2.3.9. DIC(9;20) Rearrangements

Chromosome dicentric dic(9;20) abnormality, which is a rare aberration in B-cell precursor acute lymphoblastic leukemia, was first described by Rieder et al. in 1995 [271]. It arises from the fusion of two centromeres of chromosomes 9 and 20, which leads to the loss of 9p and 20q material [271,272]. Proper diagnosis of this variant may be challenging as it can be easily omitted or mixed by the presence of other rearrangements such as monosomy 20 or del (9p) [273]. To avoid these mistakes, fluorescence in situ hybridization (FISH) should be performed instead of conventional cytogenetic analysis [274].

To date, more than 200 cases of dic(9;20) have been reported according to the Mitelman Database [275]. Epidemiological studies report a 2% frequency of dic(9;20) in children with B-ALL and less than 1% in adults, with more prevalence in women [271,272,273,275]. The peak of incidence is at 3 years of age. Five-year surveillance and overall survival are achieved by 62% and 82% of patients, respectively. Relapses in these patients are fairly common; however, treatment after that is often successful [273,276].

An et al. showed that breakpoints on the short arm of chromosome 9 target the *PAX5* locus. They also identified novel sequence partners of *PAX5* such as *ASXL1* (20q11.21), *C20ORF112* (20q11.21), and *KIF3B* (20q11.21). As for the breakpoints at 20q, they are mainly concerned with the *ASXL1* gene, causing its disruption. What is more, dic(9;20) B-ALL is also frequently associated with hetero- or homozygous losses of *CDKN2A/B* [273,277,278]. However, the breakpoints were not identical in all cases, suggesting that the final result of these aberrations is loss of genetic material rather than gene rearrangement [278,279].

Lönnerholm et al. implied the effectiveness of L-asparaginase, cytarabine, and corticosteroids in the treatment of dic(9;20) B-ALL. These outcomes were confirmed in Pichler et al.’s study, in which the 5-year event-free and overall survival were 75 ± 11% and 94 ± 6%, respectively [280,281].

The clinical and prognostic implications of dic(9;20)-related B-ALL are largely unknown. Therefore, additional series of studies are needed in order to make the best possible clinical decisions in the future.

Information on the genetic biomarkers described in the above section is presented as a summary in Table 2.

## 3. Prognostic and Therapeutic Significance

It has been noticed that mortality of ALL in Europe, the United States, and Japan has slightly decreased due to the improvement in treatment and development of new technologies [22]. The identification of new biomarkers of acute lymphoblastic leukemia, and thus a better understanding of its molecular basis, may lead to better monitoring of the course of the disease. In-depth identification of genetic aberrations in this neoplasm is crucial to assess the risk of recurrence and implement molecularly targeted therapies to reduce this risk [20]. A more accurate risk calculation will allow for better treatment of ALL with fewer side effects [180]. However, extensive screening for genetic susceptibility to leukemia is not recommended because of the potentially large false predictive value. Many children with the genetic variants peculiar to ALL will never develop it [9].

Progress in DNA sequencing and integrated analysis of multiscale biological data allowed us to discover new genetic groups and disorganized pathways in the context of ALL, which previously were not classified due to the lack of aneuploidy or single chromosomal rearrangements [20]. These new subtypes often display latent cytogenetic changes and have different gene expression profiles. Many clinically significant changes require the use of next-generation molecular methods that use RNA sequences for their detection [282].

There is a need for new research using targeted therapies in the treatment of the first-line treatment of the disease. Research is ongoing to develop new antibodies and cellular immunotherapy, but at the moment they are effective only in some patients. A thorough understanding of the entire spectrum of genetic defects opens up perspectives for potential therapeutic targeting and precision medicine in childhood ALL [180].

## Figures and Tables

**Table 2:** Characteristics of B-ALL subtypes.

Subtype	Genetic Alteration	Frequency in Childhood ALL	Frequency in Adult ALL	Prognosis	Targeted Therapy	References
High Hyperdiploidy	Aneuploidy (51–65 chromosomes)	30%	10%	Good prognosis	-	[23,25,27,28,29]
Low Hyperdiploidy	Aneuploidy (47–50 chromosomes)	10–11%	10–15%	Poor prognosis	-	[36,37]
Near Haploidy	Aneuploidy (24–31 chromosomes)	1–2%	-	Poor prognosis	Potential use of PI3K inhibitors	[28,39,40,41,42,45]
Low Hypodiploidy	Aneuploidy (32–39 chromosomes)	1–2%	4%	Poor prognosis	Potential use of PI3K inhibitors	[28,39,40,41,42,45]
High hypodiploidy	Aneuploidy (40–44 chromosomes)	2–3%	7%	Poor prognosis	Potential use of PI3K inhibitors	[38,39,40,42]
iAMP21	Amplification	1.5–2%	1%	Intermediate prognosis	-	[50,54]
*BCR*::*ABL1*	Translocation	3–4%	15–20%	Poor prognosis	TKI	[59,68,69,72]
Ph-like ALL	Gene fusions	15%	20–24%	Poor prognosis	TKI, JAK2 inhibitors, JAK1/JAK3 inhibitors, TYK2 inhibitor, Crizotinib, MEK inhibitors, FAK inhibitors, FLT3 inhibitors	[77,88,89,96,97,98,99,100,101,102,103,104,105,106]
*TCF*::*PBX1*	Translocation	6%	6%	Intermediate prognosis	Dasatinib, ruxolitinb	[82,130,135,136,139]
*TCF3*::*HLF*	Translocation	<1%	<1%	Intermediate prognosis	Venetoclax	[89,90,141,142,145]
*IKZF1*	Deletion/point mutation/gene fusion	16–27%	40–50%	Poor prognosis	-	[183,199,201]
*CRLF2*	Gene fusions/point mutation	5%	5%	Poor prognosis	Potential use of JAK inhibitors	[81,241,247,248,249,250,251]
*MEF2D*	Gene fusions	4%	4%	Poor prognosis	HDAC inhibitors, staurosporine, venetoclax	[181,254,255]
*CDKN2A*	Deletion/hypermethylation	15–35%	30–45%	Poor prognosis	CDK4/CDK6 inhibitors	[262,266,268,269,270]
*ETV6*::*RUNX1*	Translocation	25%	<5%	Good prognosis	-	[148,167,168]
*ETV6*::*RUNX1*-like	Translocation	2–3%	<1%	Poor prognosis	-	[177,180]
*KMT2A*	Translocation/inversion	5% (70–80% in infants)	10%	Poor prognosis	Dot1L, bromodomain, menin, BCL-2, polycomb repressive complex inhibitors	[108,119,127]
*DUX4*	Gene fusions	4–7%	4–7%	Good prognosis	Possibly	[130,225,228]
*PAX5*alt	Gene fusions/deletion/amplification	7–10%	8–10%	Intermediate prognosis	tyrosine kinase inhibitors (NRAS, KRAS, and FLT3)	[130,177]
*PAX5* P80R	hotspot mutation (PAX5 p.Pro80Arg mutation)	3–4%	1–4%	Intermediate prognosis	Potential use of Ras, JAK/STAT, FLT3, BRAF and PIK3CA inhibitors	[130,177]
*ZNF384*	Gene fusions	3–5%	3–8%	Intermediate prognosis	FLT3	[177,219,238]
*NUTM1*	Gene fusions	1%	-	Good prognosis	Bromodomain inhibitors	[177,257]

## Data Availability

Not applicable.
